# Case Report: Pulmonary artery banding for an older child with end-stage dilated cardiomyopathy

**DOI:** 10.3389/fped.2026.1941518

**Published:** 2026-10-12

**Authors:** Jiayuan Huang, Ling Sun, Changqing Tang, Yue Cao, Xifeng Liang, Yu Chang, Xin Li, Xin Huang, Jie Huang

**Affiliations:** 1 Department of Pediatric Cardiology, Children’s Hospital of Soochow University, Suzhou, China; 2 Department of Pediatric Cardiothoracic Surgery, Children’s Hospital of Soochow University, Suzhou, China

**Keywords:** case report, dilated cardiomyopathy, heart failure, pediatric, pulmonary artery banding

## Abstract

Therapeutic options for children with end-stage dilated cardiomyopathy are limited. Pulmonary artery banding has been proposed as a potential bridge or alternative strategy in children with severe left ventricular dysfunction and preserved right ventricular function, but related experience in older children is limited. We report a 5.5-year-old girl diagnosed with dilated cardiomyopathy who showed progressive left ventricular dilation and persistent systolic dysfunction despite long-term medical therapy. Preoperative echocardiography showed left ventricular end-diastolic diameter (LVEDD) of 64.4 mm, Z-score of +9.7, and left ventricular ejection fraction (LVEF) of 25.0%, while cardiac magnetic resonance imaging (CMR) demonstrated relatively preserved right ventricular systolic function. Pulmonary artery banding was performed without major perioperative complications. At 4-month follow-up, her clinical symptoms improved from NYHA class II to class I; however, echocardiography showed persistent reverse remodeling, with an LVEF of 25.7% and an LVEDD Z-score of +8.8. This case suggests that PAB may provide symptomatic benefit in older children with end-stage DCM but that delayed intervention may limit myocardial recovery.

## Introduction

Dilated cardiomyopathy (DCM) is the most common childhood cardiomyopathy with the annual incidence of 0.57 cases per 100,000 per year (1). Pediatric LV-DCM is a leading cause of cardiac death. However, treatment options for children with end-stage DCM are limited. Heart transplantation is recognized as the only viable life-saving option for end-stage DCM, but its long-term outcomes in children are still suboptimal (2).

Pulmonary artery banding (PAB), an old surgical procedure, has been recently proposed as a bridge or alternative strategy for DCM with low left ventricular ejection fraction but preserved right ventricular function. It is done only in a few centers worldwide and related experience in older children with long-standing left ventricular dilation is limited. Here, we present the case of a 5.5-year-old child with end-stage dilated cardiomyopathy who underwent PAB after prolonged medical therapy.

## Case report

A female child was initially admitted to hospital for “tachycardia, tachypnea and excessive sweating especially during feeding” at 4 months of age. Echocardiography demonstrated severe left ventricle (LV) dilation with markedly reduced systolic function (LVEF 28.6%), leading to a diagnosis of dilated cardiomyopathy. The patient had no prior significant medical and family history. No pathogenic variants related to cardiomyopathy were detected in whole-exome sequencing (WES). Soon afterwards, she was treated with optimized medical therapy, mainly including Captopril, Carvedilol, Furosemide, Spironolactone, Methylprednisolone and Digoxin for 6 months. Follow-up echocardiography revealed the dilated LV with Z-score of 6.3 and LVEF of 26% at 11 months old. In addition to the foregone medicines, intravenous immunoglobulin G (IVIG) and milrinone infusion were also selected during the subsequent treatment course. Unfortunately, progressive left ventricular enlargement and persistent heart failure were observed over the following years. At 5.5 years of age, the patient was readmitted to the hospital with the complaint of coughing for half a month. Given the mild limitation of physical activity and suboptimal weight gain, the patient was classified as NYHA class II. No obvious cyanosis, peripheral edema, hepatomegaly, or pulmonary rales were observed. Echocardiography showed advanced LV dilation (LVEDD 64.4 mm, Z-score +9.7) and severely impaired LVEF (25.0%), calculated using the biplane Simpson method (Figure 1A). Moderate mitral regurgitation was also present. Levosimendan was also administered at a dose of 0.05 µg/kg/min, but without improvement. Although her clinical symptoms were classified as NYHA class II at readmission, the patient was considered to have end-stage DCM because of persistent severe LV dysfunction, repeated need for inotropic support, and failure to respond to optimized medical therapy. CMR demonstrated unchanged severe LV-DCM but preserved right ventricular systolic function (EF 45%). After a multidisciplinary discussion, PAB was suggested based on previous reports and institutional experience due to progressive cardiac dilation and limited improvement in cardiac function. Full written parental consent was obtained. PAB was done with a Gore-Tex vascular graft of 4 mm diameter, fixed by 4 consecutive polypropylene 6.0 U-stitches. The tightness of the banding was guided by systemic hemodynamic status and transoesophageal echocardiographic evaluation of ventricular dimensions and the position of the interventricular septum intraoperatively. At an RV pressure of 60 mmHg (for a systemic pressure of 90 mmHg), a left-sided shift of the interventricular septum was obtained without compromising circulation. The patient returned to ICU with closed chest and need of limited hemodynamic support.

**Figure 1:**
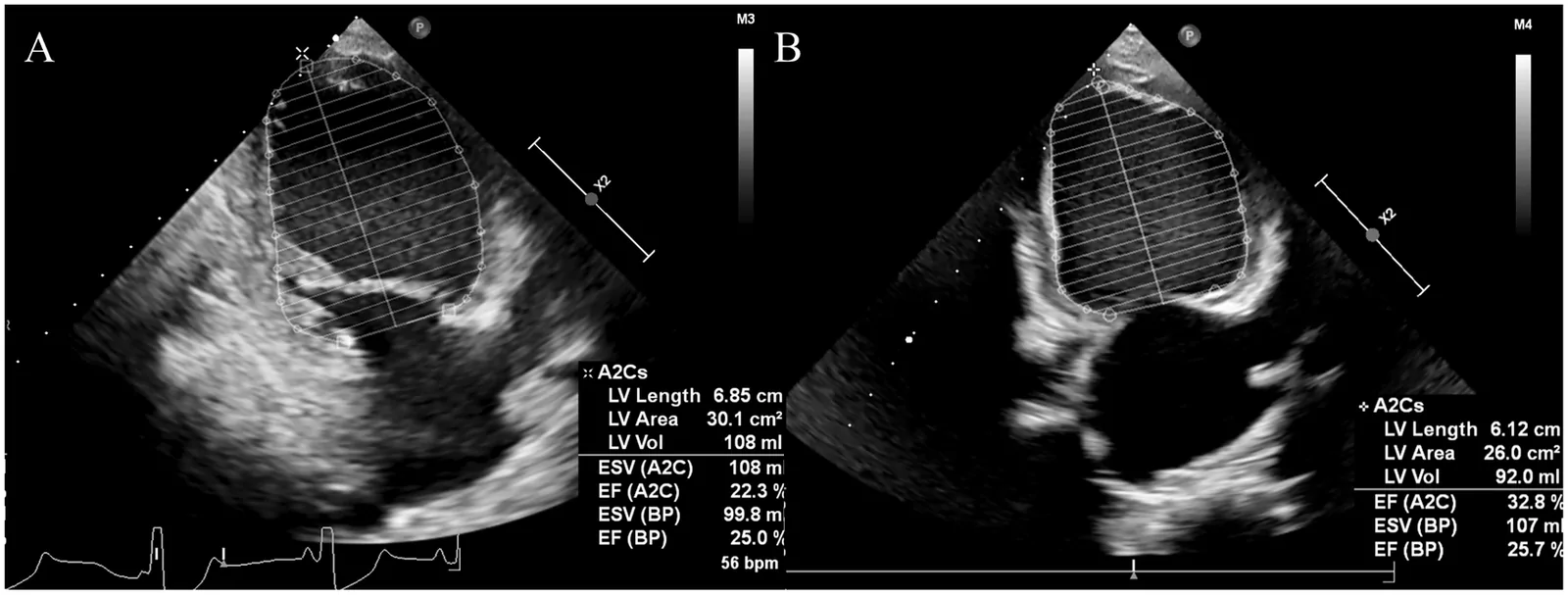
Echocardiogram before pulmonary artery banding **(A)** and at 4-month follow up **(B)**.

Postoperative recovery from the PAB was uneventful, and no major complications related to the procedure occurred. Inotropics including dopamine, milrinone and levosimendan could be completely stopped within 7 days. Then the patient was transferred to general ward and resumed the aforementioned medications until discharge. On echocardiography, a reduction in mitral regurgitation from moderate to mild was observed while LV systolic function remained poor. Clinical symptoms improved from NYHA class II to class I after surgery. At 4-month follow-up, the patient remained clinically stable while echocardiography demonstrated persistent left ventricular dysfunction with limited reverse remodeling (LVEF 25.7%, LVEDD 63.3 mm, Z-score +8.8) (Figure 1B). The clinical timeline of the patient is summarized in Table 1.

**Table 1:** Clinical timeline of the patient.

Time point	Key clinical findings	Assessment	Intervention	Outcome
4 months of age	Tachycardia, tachypnea, and excessive sweating during feeding.	Echocardiography showed severe LV dilation and reduced systolic function; LVEF 28.6%. DCM was diagnosed.	Heart failure therapy was initiated, including captopril, carvedilol, furosemide, spironolactone, methylprednisolone, and digoxin.	Persistent cardiac dysfunction.
11 months of age	Follow-up after initial treatment.	Echocardiography showed persistent LV dilation; LVEDD Z-score +6.3 and LVEF 26%.	Previous heart failure medications were continued.	No significant improvement in LV systolic function.
Following 4 years	Progressive LV enlargement and persistent heart failure during follow-up.	Serial echocardiographic follow-up suggested progressive LV dilation and sustained systolic dysfunction.	Regular IVIG and milrinone infusion were administered.	Progressive disease despite individualized medical therapy.
5.5 years of age	Coughing for half a month; NYHA class II cardiac function.	Echocardiography showed advanced LV dilation: LVEDD 64.4 mm, Z-score +9.7, and LVEF 25.0%.	Levosimendan was administered at 0.05 μg/kg/min.	No obvious improvement.
Preoperative evaluation	Progressive LV dilation and limited response to medical therapy.	CMR demonstrated severe LV-DCM with preserved RV systolic function; RVEF 45%.	PAB was recommended after MDT evaluation.	The patient was considered suitable for PAB.
Surgery	_	TEE-guided band adjustment was based on ventricular dimensions and septal position. At an RV pressure of 60 mmHg with systemic pressure of 90 mmHg, leftward septal shift was achieved.	Pulmonary artery banding.	The procedure was completed without compromising circulation.
Early postoperative period	Postoperative recovery was uneventful.	No major procedure-related complications were noted.	The aforementioned medications were continued until discharge.	Clinical symptoms improved from NYHA class II to class I.
4-month follow-up	The patient remained clinically stable.	Echocardiography showed persistent LV dysfunction with limited reverse remodeling: LVEF 25.7%, LVEDD 63.3 mm, and Z-score +8.8.	Continued follow-up and medical management.	_

CMR, cardiac magnetic resonance imaging; DCM, dilated cardiomyopathy; IVIG, intravenous immunoglobulin G; LV, left ventricle; LV-DCM, left ventricular dilated cardiomyopathy; LVEDD, left ventricular end-diastolic diameter; LVEF, left ventricular ejection fraction; MDT, multidisciplinary team; NYHA, New York Heart Association; PAB, pulmonary artery banding; RV, right ventricle; RVEF, right ventricular ejection fraction; TEE, transoesophageal echocardiography.

## Discussion

Dilated cardiomyopathy is a myocardial disorder with the characteristics of left ventricular dilation and systolic dysfunction. Myocarditis and neuromuscular disorders are the most common causes during childhood, with familial DCM, inborn errors of metabolism, and malformation syndromes less common (1). Recent studies have shown that auto-inflammatory process also plays a significant role either in triggering or aggravating the progress of DCM (3, 4). It is a leading cause of cardiac death. In particular, genetic and idiopathic DCM, have a poor outcome, especially in genetic forms when patients carry more than one variant in DCM genes (5). In this population, progression to end-stage heart failure (HF) is frequent.

The current guidelines for adults with symptomatic HF recommend combination therapy with angiotensin receptor/neprilysin inhibitors, β-blockers, mineralocorticoid receptor antagonists, and sodium-glucose cotransporter 2 inhibitors (6, 7). However, the anti-HF therapies used in DCM children are primarily based on institution-specific clinical experience and have not been verified by large controlled or randomized studies. In addition, some of studies have suggested that given immunosuppressive therapy consisting of intravenous immunoglobulin G and prednisolone could benefit ventricular function in DCM patients (8, 9). Nevertheless, some of DCM patients, including our case, still remain symptomatic with refractory HF despite maximal medical therapy.

Heart transplantation is recognized as the only viable life-saving option but usually limited by high cost, scarcity of donors and series of complications. Based on the experience of pediatric patients with congenitally corrected transposition of the great arteries in which PAB was used for re-training the sub-pulmonary left ventricle, Schranz et al. from Giessen, Germany decided to perform a central pulmonary artery banding in a DCM infant with LV failure for the first time in 2007, because PAB is an established approach for patients with a morphologic RV in systemic position to retrain the LV and to improve RV failure and ameliorate tricuspid insufficiency (10). Six weeks after banding, echocardiographic assessment revealed the LV end-diastolic diameter decrease from 41 to 30 mm with a significant improvement of systolic function (10). To summarize, PAB could induce RV stress, improve LV end-diastolic volume, pressure, and LV filling dynamics via Anrep and Frank-Starling effects, which is associated with leftward mechanical shift of the interventricular septum and the enhancement of LV contractility. Together with the reestablishment of intra- and interventricular synchrony and less left atrial congestion, PAB contributes to clinical improvement in DCM children (11–13).

In recent years, PAB has been introduced in some cardiac centers from America, Asia, and Europe for possible myocardial remodeling to improve cardiac function. Worldwide experience with PAB was consolidated in a multicenter study from 15 centers of 11 countries, showing that PAB was selectively performed without operative mortality in 61 patients between 2006 and 2017. The overall medium-term mortality is modest (8/61, 13%), while 12 patients (19%) had subsequent HTx as planned or because of PAB non-responders. Additionally, the majority of patients (34/61,56%) has experienced functional recovery and 8 patients (13%) showed improvement in LV function, which is encouraging (14). Most recently, a multicenter Europe study enrolling 31 children exhibited that there were only 4 (13%) early deaths, and at a median follow-up 1 late death and 3 HTx (10%). In addition, all 23 survivors (74%) had gradual normalization of LV function and dimensions, supporting that PAB can be an effective procedure to treat end-stage DCM children, as alternative strategy for bridging to transplant or recovery (15).

Patient selection is an important consideration. In previously reported experiences, PAB was generally considered in children with progressive heart failure despite optimized medical therapy (16), which parallels the definition of end-stage dilated cardiomyopathy in the AHA guidelines (17). In the present study, we reported a case of a 5.5-year-old end-stage DCM patient who underwent PAB after aggressive individualized HF medical treatment for 5 years. IVIG and inotropics were administered but the LV continued to progressively enlarge, with unsatisfactory improvement in cardiac function. Although the patient experienced an uneventful recovery and a notable short-term improvement in clinical symptoms to NYHA class I, her latest echocardiogram revealed that the LV had enlarged again (LVEDD 63.3 mm, Z-score +8.8) and LVEF remained low at 25.7%. Unlike the predominantly infant populations described in previous studies, where substantial LV reverse remodeling and functional recovery have been reported, this case involved an older patient with prolonged left ventricular dilation and dysfunction, a clinical feature that may influence the surgery outcome. It has been proven in the previous studies that the neonatal heart has a capacity for myocardial tissue regeneration so as to repair myocardial damage and completely recover cardiac function (18, 19). Regrettably, this cardiac regenerative capacity is age-dependent and would diminish after 1 year old (20). Schranz et al. suggested a 6-year-old age limit for PAB in DCM (11). However, the maximal age threshold for patients considered for PAB has been lowered across the years. Myers et al. described an increased risk of LV dysfunction when PAB was performed after 2 years of age (21). In most recent studies worldwide, the median age at PAB is even below 1 year old (15, 22–26). This discrepancy highlights a critical timing for myocardial recovery.

In addition to age, preoperative status may also be associated with prognosis. Spigel ZA et al. showed the result that the recovery rate was <1/3 in the United States while >2/3 in Germany; and there were >2/3 patients in the United States underwent preoperative intubation while <1/3 in Germany, highlighting the potential influence of preoperative disease severity and patient selection on outcomes (22). Furthermore, it is also reported that LVEDD with a z-value of above +5 has a low incidence of spontaneous recovery (1). In our case, LVEF remained a low level despite optimal treatment for nearly 5 years (mostly<30%), indicating a state of advanced congestive heart failure, which may be associated with limited myocardial recovery. Overall, infants (under 1 year) possess a higher degree of myocardial plasticity and regenerative capacity, making them ideal candidates for reverse remodeling via PAB. In contrast, the prolonged duration of LV dysfunction and advanced age may have limited the potential for myocardial recovery. For these older patients, PAB may be associated with clinical improvement even when substantial LV reverse remodeling is limited. Previous clinical observation has also demonstrated that hemodynamic improvement after PAB may occur despite minimal early echocardiographic changes (27). However, the specific mechanism underlying this phenomenon cannot be determined based on a single case.

This case report has several limitations. As a single case, it cannot define the optimal indications of PAB in pediatric dilated cardiomyopathy. In addition, the follow-up period was limited to 4 months, making it difficult to evaluate long-term ventricular remodeling, heart failure progression, and late complications. Therefore, this case should be interpreted as an example of potential symptomatic benefit after pulmonary artery banding in an older child with long-standing disease, rather than definitive evidence of durable myocardial recovery. Continuous monitoring and future large-scale, long-term studies are warranted to further optimize patient selection and the timing of PAB intervention.

## Conclusion

PAB may provide symptomatic improvement in older children with end-stage DCM and preserved right ventricular function. However, delayed intervention in these children with long-standing LV dilation and dysfunction may limit reverse remodeling, highlighting the importance of optimizing patient selection and surgical timing.

## Data Availability

The raw data supporting the conclusions of this article will be made available by the authors, without undue reservation.
